# Toxicity of Oils Extracted From the Arils of *Blighia sapida* (K.D. Koenig) in Wistar Rats

**DOI:** 10.1155/2024/1998836

**Published:** 2024-11-08

**Authors:** Aklesso Nabede, Haziz Sina, Mélila Mamatchi, Tiatou Souho, Batcha Ouadja, S. M. Ismaël Hoteyi, Hafiz A. Salami, Adolphe Adjanohoun, Lamine Baba-Moussa, Kou'santa Amouzou

**Affiliations:** ^1^Laboratory of Applied Agronomic and Biological Sciences, Faculty of Sciences and Technology, University of Kara, Kara, Togo; ^2^Laboratory of Biology and Molecular Typing in Microbiology, Department of Biochemistry and Cell Biology, University of Abomey-Calavi, Abomey-Calavi, Benin; ^3^Faculty of Sciences (FDS), University of Lomé, Lomé, Togo; ^4^General Management Office, National Agronomic Research Institute of Benin, Cotonou, Benin

**Keywords:** biochemical parameters, oil extracted from *Blighia sapida*, Togo, toxicity, Wistar rat

## Abstract

*Blighia sapida* oil, a substance with a rich history of use for its nutritional, therapeutic, traditional, and cosmetic benefits, was the focus of our study. We investigated the impact of consuming edible oil from *B*. *sapida* arils on Wistar rats. The crude oil from unripe arils was extracted using cold pressing and then administered to the rats. The toxicity was evaluated according to the OECD method. Notably, there were no signs of food poisoning or adverse effects on the weight and behavior of the rats treated with *B*. *sapida* oils. The LD50 of the oil was more significant than 5000 mg/kg of body weight, and hematological and biochemical parameters did not differ significantly from the control group. Rats fed with an oil-supplemented diet showed an increase in weight compared to the negative control group. No fatty deposits were found in vital organs, and consuming the oil did not affect the immune system or biochemical biomarkers. However, excessive intake of fat may have harmful effects on tissues. Our findings strongly suggest that *B*. *sapida* oil is safe for consumption within reasonable limits. The data we present here reveal that the oil derived from *B*. *sapida* is suitable for moderate consumption and may offer various health advantages, a potential that warrants further exploration.

## 1. Introduction

Edible oils are becoming increasingly popular due to their nutritional and therapeutic properties. However, it is still a challenge for biological research to ensure their safety as most of these oils are consumed without proper verification. It was warned that this can negatively impact populations' health and nutritional status, especially those in developing countries [1]. The amount and type of fatty acids ingested also affect the body's physiological response [2]. To maintain a healthy diet, reducing the consumption of saturated fats is recommended, as well as rebalancing the omega-6/omega-3 balance and favoring long-chain omega-3 fatty acids.

The diets of a specific community depend on the availability of animal and plant species and their consumption experiences [3]. Overconsumption of trans fatty acids can lead to obesity, an increased risk of cardiovascular disease, and Type 2 diabetes, as stated [4]. However, vegetable oils play a vital role in our diet by providing essential fatty acids, ensuring healthy function, and supplying and transporting fat-soluble vitamins. Lipids also contribute to the organoleptic quality of products and are used as coating and mold release agents [5].

*Blighia sapida* (K.D. Koenig), commonly known as ackee, is a native West African evergreen tree from the *Sapindaceae* family of angiosperms [6, 7]. *Blighia sapida* is a plant species with exciting medicinal and esthetic values [8]. The fruit of this plant is consumed for the nutrition of the Togolese population, and almost all parts of the plant are used in the food field and traditional medicine [9]. In a thorough analysis of ackee arilli, researchers discovered the presence of glycosides, saponins, tannins, and potentially other phytochemicals such as anthracene and cyanogenic glycosides, flavonoids, alkaloids, and triterpenes [8]. Nutritionally, the ripe ackee fruit is a good source of protein, carbohydrates, and especially lipids [10]. Several studies have reported on the fatty acid composition of the arilli of the fruit, with linoleic acid and oleic acid being the primary fatty acids present [7, 10–12]. In Togo, stands of *B. sapida* are mainly present on fertile, deep, well-drained, and calcareous soils [13]. Despite the proven nutritional value of the aril of *B*. *sapida*, this plant remains a formidable source of poisoning through the consumption of unripe fruits [3]. However, consuming unripe fruits can be poisonous due to high concentrations of hypoglycin A, leading to deaths, especially among children [14].

Despite the proven nutritional values of the aril of *Blighia sapida*, more scientific work needs to be performed in Togo on the safety of edible oils extracted from its arils. Therefore, our work aims to evaluate these oils' toxicity by assessing biochemical, hematological, and physiological markers. This exploration could be an alternative to ensuring the safety of these oils.

## 2. Materials and Methods

### 2.1. Collection and Extraction of Oil From the Arils of *Blighia sapida*

For the extraction of oil from the arils of *B*. *sapida*, the arils were harvested in three prefectures of Togo (Tône, Kozah, and Haho) from March to October 2022, months during which the arils are available. After collecting the arils, they were dried in the sun for 2 weeks and then ground into a paste using a Retsch mixer of the SM 2000/1430/Upm/Smfet type. Then, this paste was used for oil extraction by cold pressing [15]. In the end, the traces of water contained in the vegetable oil were evaporated, and the crude oil obtained was kept cool in a freezer away from light for later analyses.

### 2.2. Experimental Animals

The toxicity study involved male and female Wistar rats aged 8 to 10 weeks with an average weight of 110 ± 2 g. These rats were sourced from the animal house of the Department of Animal Physiology at the Faculty of Sciences of the University of Lomé. The animals were housed and allowed to acclimate for 1 week before the experiment. The room temperature was maintained at approximately 25°C, with a relative humidity of around 70%. The rats were fed during their active period using a 12-h light/dark cycle, and they had access to food and water *ad libitum*.

### 2.3. Ethical Consideration

The Multidisciplinary Doctoral School of the Faculty of Science and Technology (FaST) at the University of Kara (UK) approved the experimental protocol under UK/FaST/EDP/61605.

### 2.4. Acute Gavage Toxicity Study

The acute toxicity of the oil extracted from the arils of *B*. *sapida* was evaluated according to the OECD 425 method [16] in four groups of six rats each. Each group comprised subgroups of 3 males and 3 females to avoid pregnancy cases. Thus, Groups 1 and 2 represent the control groups that received physiological saline and peanut oil, respectively, with a 5000 mg/kg dose. Groups 3 and 4 received 5000 mg/kg of *B*. *sapida* ripe and unripe aril oils. The animals were weighed daily and observed during the experiment after treatment, during which these animals had free access to water and food. During this experiment, observations focused on the appearance of droppings, mobility, sensitivity to noise and pinching, feeding, and breathing [17]. To know the LD_50_, the number of deaths in each group was researched. In addition, after 14 days of observation, a blood sample from each group was taken to assay the hematological and biochemical parameters [17].

### 2.5. Repeated-Dose 28-Day Oral Toxicity

Repeated oral toxicity was evaluated in Wistar rats following the guidelines of the OECD 407 [18], considering the welfare of laboratory animals. To do this, the quantity of *B. sapida* oil contained in the different diets was evaluated by European Union standards, which recommend a proportion of 1 g/kg/day. The diets were, therefore, supplemented with two different oils (ripe and unripe aril oil) diluted at 10% in DMSO. Forty-two rats were used for the performance of this activity. These animals were divided into seven groups of six rats designated by L1 (negative control receiving physiological water), L2 (1 g/kg of ripe arils of *B*. *sapida*), L3 (1 g/kg of unripe arils of *B*. *sapida*), L4 (2 g/kg of ripe arils of *B*. *sapida*), L5 (2 g/kg of unripe arils of *B*. *sapida*), L6 (positive control receiving 1 g/kg of peanut oil), and L7 (positive control receiving 2 g/kg of peanut oil), each comprising subgroups to avoid cases of pregnancy. Furthermore, during this 28-day experiment, animals were weighed, force-fed, observed, and subjected to changing litter daily. Droppings, food consumption, and water intake were reported [19].

After 28 days of the experiment, the tested rats were fasted for 12 h, after which they were weighed and anesthetized. The blood of each rat was collected in the tubes containing EDTA as an anticoagulant and in the tubes without an anticoagulant to assay the hematological and biochemical parameters, respectively. In the end, the rats were sacrificed by decapitation, and their organs (liver, heart, organs, spleen, and lungs) were carefully removed and then preserved in 10% formalin to produce histological sections [3].

### 2.6. Evaluation of Hematological Parameters

The blood collected was used to perform a hemogram the same day using a Sysmex XN-1000 device. Thus, the levels of red blood cells, white blood cells, blood platelets, and hematocrit constants were determined according to standard methods [20].

### 2.7. Evaluation of Biochemical Parameters

The blood samples in the dry tubes were centrifuged at 2580 rpm for 15 min. The sera collected and stored at −20°C were used to assay biochemical markers of specific vital organs (liver, kidneys, heart, spleen, and lungs) using the Sigma Chemical Company Kits (St Louis, Missouri, USA). Thus, the enzymatic method assayed transaminases [17]. Total bilirubin and conjugated bilirubin were determined by a colorimetric method and total cholesterol, HDL-cholesterol, LDL-cholesterol, triglycerides, urea, blood sugar, creatinine, and uric acid were determined by the enzymatic and colorimetric methods [3]. Finally, the electrolytes (calcium, magnesium, and phosphorus) were measured by the colorimetric method using an electrolyte analyzer [19].

### 2.8. Determination of Some Parameters Relating to Animal Physiology

#### 2.8.1. Measurement of Relative Masses of Organs

After 28 days of experimentation, the vital organs (heart, liver, kidneys, spleen, and lungs) were immediately removed and washed in a saline solution before weighing them. Finally, using the formula described by Bohué et al. [5], the relative masses of the liver, heart, kidney, lung, and spleen were determined as

(1) $$\begin{array}{c} \text{relative mass of the heart }\left(\text{rHM}\right):\;rHM=\left(\frac{\text{mass of the heart of the sacrificed animal}}{\text{mass of the sacrificed animal}}\right)\times 100, \end{array}$$

(2) $$\begin{array}{c} \text{relative liver mass }\left(\text{rLiM}\right):\text{ rLiM}=\left(\frac{\text{liver mass of the sacrificed animal}}{\text{mass of the sacrificed animal}}\right)\times 100, \end{array}$$

(3) $$\begin{array}{c} \text{relative kidney mass }\left(\text{rKM}\right):\text{ rKM}=\left(\frac{\text{kidney mass of the sacrificed animal}}{\text{mass of the sacrificed animal}}\right)\times 100, \end{array}$$

(4) $$\begin{array}{c} \text{relative lung mass }\left(\text{rLuM}\right)\;:\text{ rLuM}=\left(\frac{\text{lung mass of the sacrificed animal}}{\text{mass of the sacrificed animal}}\right)\times 100, \end{array}$$

(5) $$\begin{array}{c} \text{relative spleen mass }\left(\text{rSM}\right):\text{ rSM}=\left(\frac{\text{spleen mass of the sacrificed animal}}{\text{mass of the sacrificed animal}}\right)\times 100. \end{array}$$

#### 2.8.2. Digestibility Measurement

The digestibility index (DI) was determined using the amount of digested food. To do this, we collected the animals' droppings daily from 8 a.m. to 9 a.m. Once collected, the excreta of each group were dried (20°C for 24 h) and weighed. The formula of Doh et al. [19] is used to calculate the DI as follows:

(6) $$\begin{array}{c} \text{DI}=\frac{\text{quantity of food consumed}-\text{mass of droppings produced during }a\text{ period}}{\text{weight gain during the same period}}\times 100. \end{array}$$

#### 2.8.3. Determination of Average Weight Gain (WG), Consumption Index (CI), and Hydration Index (HI)

The influence of *Blighia sapida* oil on the evolution of animal weight was investigated by daily weighing between 8 and 9 a.m. Thus, the average WG was determined by the following formula [5]: WG = mass of the animal at a given period—initial mass.

In addition, the CIs and HIs were determined daily for the group by using the formulas used by Bohué et al. [5] as

(7) $$\begin{array}{c} CI=\frac{\text{amount of food consumed during }a\text{ period}}{\text{weight gain during the same period}}, \end{array}$$

(8) $$\begin{array}{c} IH=\frac{\text{amount of water consumed during }a\text{ period}}{\text{weight gain during the same period}}. \end{array}$$

### 2.9. Data Processing and Statistical Analysis

Data were coded and inputted into Excel 2016 databases before being analyzed using SPSS and GraphPad Prism 8 software. Results are presented as a mean with standard deviation, expressed in tables and graphs. Fisher's grouping test was used to structure the means. Statistical differences were determined with *p* < 0.05.

## 3. Results

### 3.1. Evaluation of Acute Toxicity

No toxicity was observed during the 14 days of experimentation after force-feeding. The weight of the animals continually increased. Furthermore, until the end of administering the various oils extracted from *B*. *sapida*, no death was notified in the treated animals, which did not allow the determination of the LD_50_. This LD_50_ is more significant than 5000 mg/kg of body weight. The results relating to the hematological did not show any significant difference between the animals force-fed with *B*. *sapida* oil and those of the control groups (Table 1).

### 3.2. Study of the Subacute Toxicity of the Oil Extracted From the Arils of *Blighia sapida*

#### 3.2.1. Evaluation of Behavioral Parameters and Surviving Animals

During the 28 days of experimentation, observation of behavior throughout the study period made it possible to realize that no death was observed. In addition, compared to the control groups, the animals force-fed with the oil extracted from the arils of *B*. *sapida* did not show any apparent signs of toxicity. Furthermore, macroscopic observation of droppings during the experiment revealed no abnormalities, such as bloody droppings or cases of diarrhea.

#### 3.2.2. Effect of Diets on Change in Body Weight

The change in weight in male and female rats showed an overall increase over time, regardless of the group considered. However, this weight increase was significantly higher in the rats force-fed with an oil-supplemented diet than in the negative control group (*p* < 0.05). Furthermore, Table 2 illustrates the average WG in animals obtained from the average body weight of each group of rats at the beginning and end of the experiment.

#### 3.2.3. Effect of Oil Extracted From *Blighia sapida* on the Relative Weight of Harvested Organs as a Function of the Dose

The experiment was conducted for 28 days, but unfortunately, there was no significant difference in body weight between the control group and the group treated with various oils (*p* > 5%). However, it is worth noting that there was a slight increase in the liver of animals who were force-fed with peanut oil (2 g/kg), oil from ripe arils (2 g/kg), and oil from unripe arils (2 g/kg), although the increase was not statistically significant. Refer to Table 3 for detailed results.

#### 3.2.4. Determination of Zootechnical Parameters Relating to Animal Physiology

Table 4 presents the HIs for this experiment's experimental and control groups during force-feeding. The analysis reveals that water consumption did not significantly affect WG (*p* > 5%). The diets' impact on WG and food consumption suggests that the consumption of different edible oils does not affect average WG, except for the oil extracted from the unripe arils of *B*. *sapida* (2 g/kg), which significantly influenced WG (*p* < 0.05) compared to control rats. In addition, the DIs of the control and experimental groups did not significantly affect the gain and emission of droppings (*p* > 5%).

#### 3.2.5. Effect of Diets on Hematological Parameters

Table 5 reveals no significant differences in the levels of red blood cells, hemoglobin, and hematocrit between the rats that received *Blighia sapida* oil and those in the control group (*p* > 5%). However, female rats given a high oil concentration (2 g/kg) showed a slight decrease in these parameters, although the decline was not statistically significant. The hematometra constants (MCV, TCMH, and MCHC) did not reveal any significant differences among the groups (*p* > 5%). Interestingly, the aril oil did not appear to have a dose-dependent effect on the erythrocyte lineage.

It is worth noting that Table 5 demonstrates a significant increase in white blood cells in rats that were administered with ripe aril oil (1 g/kg) as compared to the other groups (*p* < 0.05). Furthermore, the study found that the oil extracted from the arils of *Blighia sapida* reduced blood platelets among female rats. However, the decrease was not statistically significant compared to the control group (Table 5).

#### 3.2.6. Effect of Diets on Biochemical Parameters

Table 6 illustrates the mean values of transaminases and bilirubin in experimental and control rats. Analysis of these results showed no statistically significant difference in transaminases (ALAT and ASAT); however, a nonsignificant reduction (*p* > 5%) of these transaminases was observed overall in male rats having consumed oil from ripe arils (1 g/kg). The absence of significance (*p* > 5%) concerning bilirubin between the control and experimental groups was recorded.

**Figure 1:**
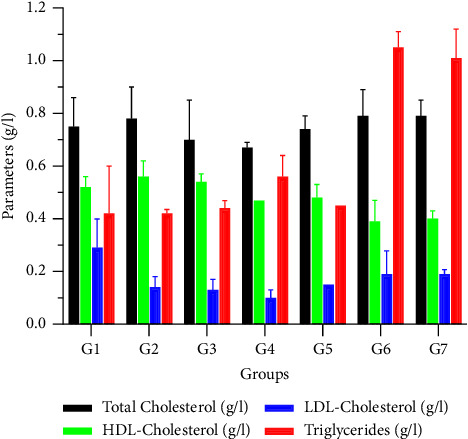
Effect of diet on total cholesterol, HDL-cholesterol, LDL-cholesterol, and triglycerides. G1: Group 1 (control); G2: Group 2 (1 g/kg of oil from ripe arils); G3: Group 3 (1 g/kg of oil from unripe arils); G4: Group 4 (2 g/kg of oil from ripe arils); G5: Group 5 (2 g/kg of oil from unripe arils); G6: Group 6 (1 g/kg of oil from peanut); G7: Group 7 (2 g/kg of oil from peanut).

The data in Figure 1 indicate that there was no change in the total cholesterol level in all force-fed animals (*p* > 5%). The data for HDL-cholesterol after 28 days show no significant difference between control and experimental rats. However, rats that received a 1 g/kg dose of oils from the arils of *B. sapida* showed a nonsignificant increase in plasma HDL-cholesterol compared to those that received peanut oil. The results for the LDL-cholesterol levels show that rats whose diets were supplemented with oils had reduced LDL-cholesterol levels compared to the negative control group, but this decrease was not significant (*p* > 5%). Finally, the results for the triglyceride levels of the control and treated animals at the end of the experiment showed that the consumption of oil extracted from the arils of *B. sapida* did not affect the triglyceride profile in the animal groups. However, a nonsignificant decrease in these parameters was reported in rats fed with *B. sapida* aril oil.

##### 3.2.6.1. Effect of Diets on Renal Biomarkers (Urea, Uric Acid, and Creatinine)

**Figure 2:**
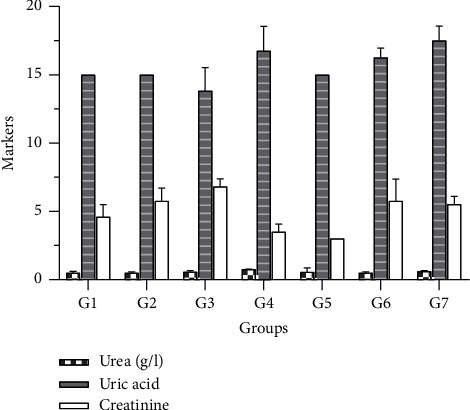
Effect of diets on urea, creatinine, and uric acid production. G1: Group 1 (control); G2: Group 2 (1 g/kg of oil from ripe arils); G3: Group 3 (1 g/kg of oil from unripe arils); G4: Group 4 (2 g/kg of oil from ripe arils); G5: Group 5 (2 g/kg of oil from unripe arils); G6: Group 6 (1 g/kg of oil from peanut); G7: Group 7 (2 g/kg of oil from peanut).

Based on the information presented in Figure 2, it appears that this oil does not hurt the renal system. However, when given at high doses (2 g/kg), the oils extracted from the arils of *Blighia sapida* led to a minor decrease in creatinine levels compared to the control groups. In addition, when administered at a dose of 1 g/kg, the oil extracted from the unripe arils of *Blighia sapida* resulted in a slight reduction in uric acid levels, although this was not significant.

##### 3.2.6.2. Effect of Diets on Serum Glucose

**Figure 3:**
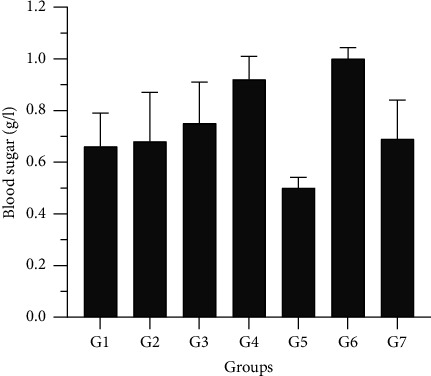
Effect of diets on blood sugar. G1: Group 1 (control); G2: Group 2 (1 g/kg of oil from ripe arils); G3: Group 3 (1 g/kg of oil from unripe arils); G4: Group 4 (2 g/kg of oil from ripe arils); G5: Group 5 (2 g/kg of oil from unripe arils); G6: Group 6 (1 g/kg of oil from peanut); G7: Group 7 (2 g/kg of oil from peanut).

The data in Figure 3 show the impact of consuming oil from the arils of *Blighia sapida* on the serum glucose levels of force-fed Wistar rats. Results indicate that this oil does not have any adverse effects on the glycemia of the control and experimental rats. However, rats treated with oil from unripe arils of *Blighia sapida* (2 g/kg) experienced a hypoglycemic effect. This hypoglycemia was insignificant when comparing the control and experimental groups.

##### 3.2.6.3. Effect of Diets on Serum Electrolytes (Calcium, Magnesium, and Phosphorus)

The results of the blood ionogram are shown in the Table 6. These results do not show any significant difference (*p* > 5%) between the different groups of control and experimental rats after gavage for 28 days.

#### 3.2.7. Histopathological Study of Vital Organs Removed (Heart, Liver, Lungs, Spleen, Kidneys)

**Figure 4:**
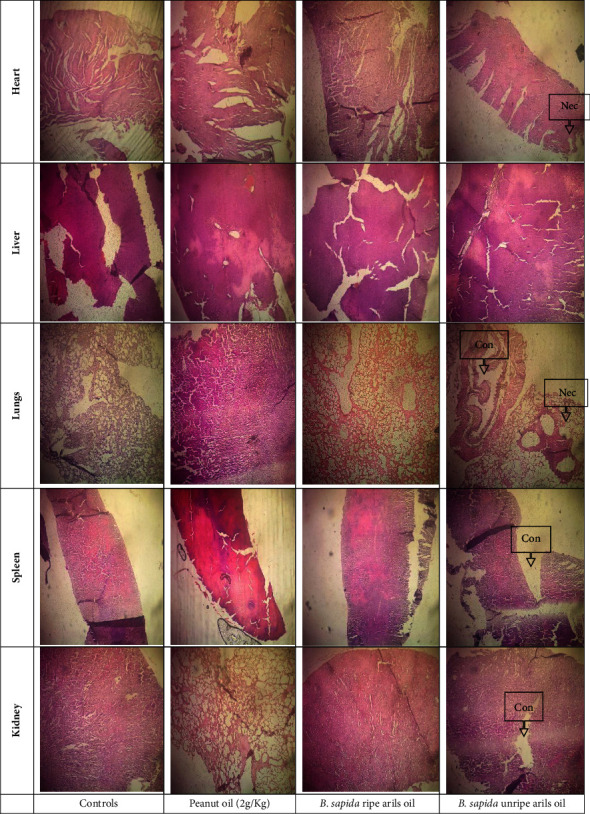
Pictures showing the histological sections of five organs (kidney, spleen, lungs, liver, and heart) removed from the tested rats (magnification: ×100; color: Hematein–eosin); Con: congestion; Nec: necrosis.

After conducting a 28-day experiment, we examined the vital organs of female subjects for abnormalities such as necrosis, fibrosis, and congestion. We found no visible abnormalities when examining the organs with the naked eye. In addition, our microscopic examination found no structural abnormalities in the control group or those who received 2 g/kg of ripe aril oil (Figure 4). However, we did observe cases of necrosis and fibrosis in females who were given a high dose of oil from the unripe arils of *Blighia sapida* (2 g/kg).

## 4. Discussion

Humans need to use edible oils to enhance the taste of our food. However, some debate has been about their potential adverse effects on our bodies. A new study is investigating the quality of oils extracted from the arils of *B*. *sapida* by testing them for toxicity in Wistar strain rats. To assess the safety of these oils on vital organs such as the liver, kidneys, lungs, heart, and spleen, as well as on eating habits and metabolic parameters, a study was conducted using a force-feeding technique. As previously noted, researchers commonly use this technique to determine product acceptability and tolerance [21]. The oil extracted from the arils of *B*. *sapida* administered orally at a dose of 5000 mg/kg of body weight for 14 days did not cause any deaths throughout the experiment. This oil, therefore, does not present any acute toxicity at this dose, according to the classification of OECD 425. Moreover, after administration of a single dose of 5000 mg/kg to rats, these oils did not modify the physiology of the treated animals.

The findings of this study revealed no food-related toxicity or mortality indications, indicating that moderate oil consumption did not lead to gastrointestinal complications or hastened digestive processes. Doh et al. [19] also noted similar observations when testing oil derived from *A. paniculata*. The study found that rats who consumed *B*. *sapida* oil did not experience any significant differences in body mass or vital organ masses compared to those who consumed peanut oil. This suggests that moderate oil consumption did not lead to obesity in those organs. Increased liver weight may be due to increased demand for detoxification or cell mass. The oil can be considered edible, but overconsumption could be harmful. The effects of consuming this oil in rats would depend on its lipid profile, as shown in a study conducted in Togo [22]. The comparison of the means of the parameters of the leukocyte lineage between the different groups carried out shows a significant increase in white blood cells in the rats treated with the oil extracted from the ripe arils (1 g/kg) compared to the other groups; this result indicates that the moderate consumption of this oil improves the immune system and does not cause anemia.

We looked for ways to measure the effects of force-feeding on animals by analyzing their diet. Based on our findings, all the oils except for unripe aril oil (2 g/kg) are safe for humans to consume. These results supported the research of Bohué et al. [5]. The toxicity of oils extracted was conducted from the arils of *B*. *sapida* on vital organs by analyzing some biochemical biomarkers. The analysis showed no significant difference in the average transaminases between experimental and control groups. However, we observed a nonsignificant reduction in these enzymes in male rats fed oil from ripe arils (1 g/kg). This suggests that the consumption of this oil does not cause the rats to experience any deterioration in the function of their liver cells, which could lead to an abnormal release of these enzymes in the blood [19]. The reduction in the level of transaminases by the oil extracted from ripe arils (1 g/kg) could be explained by its hepatoprotective effect. These findings are consistent with those of Ouattara et al. [3] on the same oils. Our analysis also showed no significance between the control and experimental groups regarding bilirubin levels (*p* > 5%). This suggests that ingesting the oil did not destroy the liver or red blood cells (hemolytic anemia) [23]. The blood sugar dosage revealed that the oil did not affect blood glucose. This explains why moderate oil consumption does not affect the metabolism of this substrate [24]. However, overconsumption of oil extracted from unripe arils of *Blighia sapida* induced hypoglycemia in force-fed rats. This is due to the presence of hypoglycin A, a toxic substance found in the unripe arils of this plant species [25, 26]. Some authors observed similar toxicity in unripe arils [27].

Liver damage can cause health problems characterized by high levels of transaminases, bilirubin, and blood glucose, indicating hepatotoxicity [28]. However, analysis of total cholesterol levels showed no significant differences between control and experimental groups, suggesting that consuming *B*. *sapida* aril oil does not contribute to metabolic syndromes. In addition, after a 28-day experiment, the HDL-cholesterol lipid fraction remained stable in both control and experimental rats. At a dose of 1 g/kg, the oil from ripe *B*. *sapida* arils showed a nonsignificant increase in blood HDL-cholesterol levels compared to control groups. This indicates that moderate consumption of ripe aril oil can protect the cardiovascular system. Furthermore, rats supplemented with oil showed a nonsignificant decrease in LDL-cholesterol levels, suggesting that these edible oils do not contribute to cholesterol deposits in blood vessels. Similar results were obtained by Ouattara et al. [3] in Côte d'Ivoire. The lack of significance in triglyceride levels between groups confirms these oils' protective role on the cardiovascular system and a lipid profile reasonably close to these oils. The liver synthesizes and excretes cholesterol, and obstruction of this organ can increase plasma cholesterol levels and lead to its deposit in arteries [29, 30].

The kidneys play a crucial role in maintaining balance by eliminating harmful substances. In cases of renal failure, the levels of these nitrogenous compounds in the bloodstream increase beyond normal levels [3]. This study noted no significant difference in the serum electrolytes (calcium, magnesium, and phosphorus) between the control and experimental groups. This finding indicates that consuming oils extracted from the arils of *B*. *sapida* does not affect the metabolism of these serum electrolytes. Similarly, research reported that certain oils did not harm the kidneys' ability to filter biomarkers such as urea, uric acid, and creatinine. Monitoring the levels of these biomarkers can provide insight into the overall health of the kidneys [31–33]. Therefore, these oils do not cause any changes in vital processes, such as the construction of human tissues and the regulation of vital reactions involving ions as cofactors [34, 35]. The histological sections indicated that moderate consumption of these oils did not harm the structure of organ tissues. However, excessive intake of these oils may lead to necrosis and fibrosis.

## 5. Conclusion

The study investigated how consuming oil from *B*. *sapida* arils affects physiological, hematological, and biochemical parameters. The results suggest that moderate consumption of this oil is similar to peanut oil, commonly used in Togo. Furthermore, the study found no evidence of toxicity associated with *B. sapida* oil consumption, as confirmed by examining vital organs. Based on these findings, ackee arilli oils can be introduced into animal and human feed as a source of triglycerides. However, excessive consumption of this oil could have adverse effects. Investigating the impact of the long-term use of vegetable oil would be interesting.

## Figures and Tables

**Table 1:** Hematological parameters of the force-fed rats.

Settings	Control	Peanut oil	Ripe aril oil	Unripe aril oil
Lymphocytes (%)	76.5 ± 2.1	87.0 ± 1.0	72.7 ± 7.7	71.2 ± 1.2
Monocytes (%)	4.0 ± 0.0	3.8 ± 1.6	5.5 ± 0.0	5.0 ± 1.7
Polymorphonuclear neutrophil (%)	23.0 ± 1.4	20.0 ± 2.7	23.5 ± 7.9	22.5 ± 4.2
Polymorphonuclear eosinophil (%)	0.0 ± 0.0	0.0 ± 0.0	0.0 ± 0.0	0.0 ± 0.0
Polynuclear basophil (%)	0.0 ± 0.0	0.0 ± 0.0	0.0 ± 0.0	0.0 ± 0.0
White blood cells (10^3^/μL)	12.6 ± 3.0	12.3 ± 3.3	13.0 ± 1.0	12.5 ± 2.1
Red blood cells (10^6^/μL)	7.5 ± 0.9	7.4 ± 0.7	7.6 ± 0.5	8.0 ± 0.9
Hemoglobin (g/dL)	10.8 ± 7.2	11.9 ± 2.3	11.6 ± 1.1	10.5 ± 0.0
Hematocrit (%)	35.3 ± 1.9	38.3 ± 0.4	38.7 ± 0.8	35.8 ± 1.1
MCV (fL)	53.4 ± 0.7	54.5 ± 1.5	55.7 ± 0.8	55.7 ± 0.1
TCMH (pg)	16.6 ± 2.0	16.3 ± 0.8	17.2 ± 0.5	16.8 ± 0.0
MCHC (g/dL)	24.8 ± 0.8	31.5 ± 0.5	29.3 ± 1.8	30.8 ± 0.6
Platelets (10^3^/μL)	737.0 ± 0.3	766.0 ± 1.6	726.2 ± 0.1	731.8 ± 3.8

*Note:* The values represent the mean ± SEM of the hematological parameters of each group, *n* = 6 rats. The analysis of variance compares the means with one factor.

Abbreviations: ACHC, average corpuscular hemoglobin content; MCHC, mean corpuscular hemoglobin concentration; MCV, mean globular volume.

**Table 2:** Data on the mean weight gain.

	Initial mass	Final mass	Average weight gain
Group 1 (control)	100.8 ± 1.1	140.7 ± 0.9	39.83
Group 2 (HAM 1 g/kg)	109.5 ± 0.9	160.2 ± 1.1	50.66^∗^
Group 3 (HAI 1 g/kg)	106.2 ± 1.3	158.0 ± 0.1	52.16^∗^
Group 4 (HAM 2 g/kg)	110.0 ± 1.4	167.5 ± 1.1	57.5^∗^
Group 5 (HAI 2 g/kg)	103.3 ± 1.4	168.5 ± 0.4	65.17^∗^
Group 6 (peanut oil 1 g/kg)	78.0 ± 0.8	130.0 ± 1.5	52^∗^
Group 7 (peanut oil 2 g/kg)	101.5 ± 1.5	166.0 ± 3.5	64.5^∗^

*Note:* HAM, oil from ripe arils; HAI, oil from unripe arils. The values represent the mean ± SEM of the weight masses of each group, *n* = 6 rats. The analysis of variance carried out the comparison of the means with one factor.

⁣^∗^*p* < 0.05.

**Table 3:** The relative weight of the organs according to the dose.

	Relative mass				
	Liver	Heart	Lungs	Spleen	Kidney
Group 1 (control)	4.4 ± 0.1	0.45 ± 0.2	0.7 ± 0.1	0.4 ± 0.2	0.8 ± 0.1
Group 2 (HAM 1 g/kg)	4.4 ± 0.8	0.47 ± 0.1	0.7 ± 0.3	0.4 ± 0.1	0.8 ± 0.1
Group 3 (HAI 1 g/kg)	4.3 ± 0.4	0.45 ± 0.4	0.7 ± 0.1	0.4 ± 0.1	0.79 ± 0.2
Group 4 (HAM 2 g/kg)	4.6 ± 1.0	0.5 ± 0.1	0.7 ± 0.1	0.4 ± 0.1	0.8 ± 0.1
Group 5 (HAI 2 g/kg)	4.7 ± 0.3	0.5 ± 0.1	0.7 ± 0.1	0.4 ± 0.1	0.8 ± 0.1
Group 6 (peanut oil 1 g/kg)	4.3 ± 0.1	0.5 ± 0.1	0.7 ± 0.1	0.4 ± 0.1	0.8 ± 0.1
Group 7 (peanut oil 2 g/kg)	4.6 ± 1.4	0.5 ± 0.1	0.7 ± 0.2	0.4 ± 0.1	0.8 ± 0.4

*Note:* HAM, oil from ripe arils; HAI, oil from unripe arils. The values represent the mean ± SEM of the relative masses of each group.

**Table 4:** The determined indices.

	Hydration index	Consumption index	Digestibility index
Group 1 (control)	3.6 ± 0.2	1.5 ± 0.4	0.8 ± 0.1
Group 2 (HAM 1 g/kg)	3.8 ± 0.4	1.6 ± 1.1	0.8 ± 0.1
Group 3 (HAI 1 g/kg)	4.8 ± 0.8	1.2 ± 0.4	0.8 ± 0.5
Group 4 (HAM 2 g/kg)	3.7 ± 1.4	1.3 ± 0.5	0.7 ± 0.1
Group 5 (HAI 2 g/kg)	3.4 ± 0.8	0.5 ± 0.6⁣^∗^	0.8 ± 0.2
Group 6 (peanut oil 1 g/kg)	3.6 ± 0.9	1.2 ± 0.5	0.8 ± 0.4
Group 7 (peanut oil 2 g/kg)	3.6 ± 0.9	1.2 ± 0.9	0.7 ± 0.1

*Note:* HAM, oil from ripe arils; HAI, oil from unripe arils. The values represent the mean ± SEM of each group's food control indices, *n* = 6 rats. The analysis of variance carried out the comparison of the means with one factor.

⁣^∗^*p* < 0.05.

**Table 5:** Hematological parameters of the different groups of force-fed rats.

	Group 1 (control)	Group 2 (HAM 1 g/kg)	Group 3 (HAI 1 g/kg)	Group 4 (HAM 2 g/kg)	Group 5 (HAI 2 g/kg)	Group 6 (peanut 1 g/kg)	Group 7 (peanut 2 g/kg)
Lymphocytes (%)	71.5 ± 5.0	74.4 ± 6.0	71.0 ± 7.7	72.2 ± 5.2	70.3 ± 6.3	72.7 ± 1.6	70.5 ± 0.6
Monocytes (%)	1.0 ± 0.0	1.3 ± 0.6	1.0 ± 0.0	1.5 ± 0.7	1.0 ± 0.0	2.0 ± 0.7	2.0 ± 0.9
PN (%)	27.7 ± 4.4	27.8 ± 6.7	28.5 ± 7.9	26.5 ± 4.7	24.7 ± 6.5	26.3 ± 0.2	23.3 ± 0.7
Eosinophils (%)	1.0 ± 0.0	1.0 ± 0.0	1.0 ± 0.0	1.0 ± 0.0	1.0 ± 0.0	1.0 ± 0.0	1.0 ± 0.6
Polynuclear basophils (%)	00.0	00.0	00.0	00.0	00.0	00.0	0.0
White blood cells (10^3^/μL)	12.1 ± 4.0	15.3 ± 3.8^∗^	11.6 ± 4.9	12.8 ± 2.9	11.6 ± 2.2	11.0 ± 0.7	12.3 ± 0.8
Red blood cells (10^6^/μL)	8.3 ± 0.9	8.4 ± 0.6	7.9 ± 0.9	7.16 ± 0.6	7.8 ± 3.2	8.5 ± 1.6	6.8 ± 0.2
Hemoglobin (g/dL)	12.8 ± 5.17	13.6 ± 2.3	12.6 ± 1.1	10.5 ± 0.9	10.2 ± 4.1	12.6 ± 2.1	11.7 ± 0.3
Hematocrit (%)	42.3 ± 4.5	43.4 ± 0.8	42.4 ± 0.8	40.7 ± 1.2	40.4 ± 4.1	43.5 ± 2.8	40.0 ± 0.2
MCV (fL)	51.2 ± 1.0	51.5 ± 1.5	52.7 ± 0.8	51.7 ± 0.7	52.5 ± 0.9	57.2 ± 0.1	54.5 ± 0.6
TCMH (pg)	15.8 ± 2.0	16.2 ± 0.8	15.7 ± 1.5	16.3 ± 0.9	16.3 ± 0.7	16.9 ± 2.5	16.1 ± 0.3
MCHC (g/dL)	29.0 ± 0.8	31.5 ± 0.5	29.7 ± 1.1	30.6 ± 0.9	31.0 ± 1.1	29.7 ± 2.1	29.9 ± 1.4
Platelets (10^3^/μL)	923.3 ± 0.7	926.0 ± 88.6	943.2 ± 22.2	861.8 ± 163.2	874.3 ± 189.3	855.3 ± 1.1	879.0 ± 0.5

*Note:* HAM, oil from ripe arils; HAI, oil from unripe arils. The values represent the mean ± SEM of the various hematological parameters of each group, *n* = 6 rats. The means were compared by one-way analysis of variance.

Abbreviation: PN, polymorphonuclear neutrophils.

⁣^∗^*p* < 0.05.

**Table 6:** Biochemical parameters of the force-fed rats.

	Control	Peanut oil	HAM	HAI
Urea (g/L)	0.5 ± 0.4	0.4 ± 0.8	0.53 ± 0.1	0.6 ± 0.1
Blood sugar (g/L)	1.3 ± 0.3	1.2 ± 0.1	1.3 ± 0.1	1.0 ± 1.7
Creatinine (mg/L)	5.0 ± 0.8	5.0 ± 0.5	5.0 ± 1.5	5.0 ± 0.2
Uric acid (mg/L)	15.0 ± 0.0	15.0 ± 0.5	15.0 ± 1.7	15.0 ± 0.9
Total cholesterol (g/L)	0.6 ± 0.2	0.6 ± 0.1	0.7 ± 0.1	0.7 ± 0.3
HDL (g/L)	0.3 ± 0.04	0.3 ± 0.3	0.4 ± 0.1	0.4 ± 0.4
LDL (g/L)	0.1 ± 0.1	0.2 ± 0.1	0.1 ± 0.1	0.1 ± 0.1
VLDL (g/L)	0.1 ± 0.1	0.1 ± 0.1	0.1 ± 0.1	0.2 ± 0.1
Triglycerides (g/L)	0.5 ± 0.1	0.5 ± 0.1	0.5 ± 0.1	0.6 ± 0.2
ASAT (IU/L)	156.8 ± 0.4	167.0 ± 0.8	165.0 ± 2.2	158.0 ± 0.1
ALAT (IU/L)	88.0 ± 2.4	88.0 ± 1.0	78.2 ± 0.8	92.4 ± 0.3
Total bilirubin (mg/L)	3.0 ± 1.2	3.0 ± 0.8	3.0 ± 0.3	3.0 ± 0.5
Direct bilirubin (mg/L)	2.0 ± 0.1	2.0 ± 0.2	2.0 ± 0.4	2.0 ± 0.3
Ca^2+^ (mg/L)	108.0 ± 0.1	111.5 ± 0.8	107.0 ± 0.6	109.2 ± 0.3
Mg^2+^ (mg/L)	16.0 ± 0.5	15.8 ± 0.4	15.5 ± 0.2	16.5 ± 0.1
Phosphorus (mg/L)	52.0 ± 0.5	55.0 ± 0.1	54.0 ± 0.2	54.0 ± 0.6

*Note:* HAM, oil from ripe arils; HAI, oil from unripe arils. The values represent the mean ± SEM of the biochemical parameters of each group, *n* = 6 rats. The analysis of variance carried out the comparison of the means with one factor.

## Data Availability

The data supporting the findings of this study are available from the corresponding author upon request.
